# Prescribing patterns of rural family physicians: a study in Kermanshah Province, Iran

**DOI:** 10.1186/s12889-017-4932-1

**Published:** 2017-11-28

**Authors:** Fariba Ahmadi, Ehsan Zarei

**Affiliations:** 1 Deputy of Health Affairs, Kermanshah University of Medical Sciences, Kermanshah, Iran; 2 Department of Public Health, School of Health, Shahid Beheshti University of Medical Sciences, Velenjak, Tehran, Iran

**Keywords:** Irrational use of drugs, Prescribing behavior, Family physician, Primary health care

## Abstract

**Background:**

The inappropriate use of drugs due to irrational prescriptions is a common problem in Iran, but there is little evidence of prescription patterns in rural family physicians. This study aimed to explore the prescribing pattern and rational drug use indicators for family physicians using Index of Rational Drug Prescribing (IRDP) in Kermanshah Province, Iran.

**Methods:**

In this retrospective study, 352,399 prescriptions from 184 family physicians in 103 primary health care (PHC) centers were examined. As stated, an analysis was done for rational use indicators suggested by World Health Organization (WHO): e.g., the percentage of prescriptions containing antibiotics, injections, and those prescribed by a generic name and from a national essential medicine list, plus the average number of drugs per prescription; these factors were all taken into account. Rational drug use was studied with the IRDP.

**Results:**

The average number of drugs per prescription was 3.14 (± 1.2) and the average cost per prescription was 116,740 IRR (USD 3.6). Around 19% of prescriptions had more than four drugs, while the percentage of prescriptions involving antibiotics and injections was 52.1% and 24.4%, respectively. There was 95.1% drugs prescribed by their generic name and 95.9% were retrieved from the essential drugs list. The value of the IRDP was 3.70 out of 5.

**Conclusion:**

The findings of this study showed that some degree of irrational drug prescribing exists among family physicians, especially in terms of injections, antibiotics, and polypharmacy. It is recommended that there be continuing education programs for physicians regarding rational prescribing for different kinds of medical indications. Clinical practice guidelines should also assist with the rational use of medicine.

## Background

Medications play an important role in health and can help cure disease and relieve pain, if properly used [1]. Drugs are expensive and account for 25% of all health care expenditures [2], thus, their rational and efficient use is essential. The appropriate use of medicines can achieve better and safer health care for patients and communities [3, 4].

The World Health Organization (WHO) has defined the rational use of drugs as “patients receive medications appropriate to their clinical needs, in doses that meet their own individual requirements, for an adequate period of time, and at the lowest cost to them and their community” [5]. Therefore, in collaboration with the International Network for Rational Use of Drugs (INRUD), the WHO has developed a set of indicators for measuring the rational use of drugs in primary health care settings and enable comparisons between health facilities including the average number of drugs prescribed per prescription, the percent of drugs prescribed by generic name, the percent of drugs prescribed from the essential medicines list, the percent of prescriptions containing an antibiotic, and the percent of prescriptions containing an injection [6].

The World Bank estimates that 20–50% of healthcare costs in developing countries are spent on medicines and medical equipment [4]. The WHO estimates that about half of the prescribed medicines are used irrationally [1, 7, 8]. Inappropriate, inefficient, and uneconomic use of drugs is a serious problem and usually occurs in healthcare facilities. This ultimately leads to adverse effects for patients [4, 8, 9]. Irrational drug use includes over- and under-prescribing, polypharmacy, lack of indication, inappropriate use of antibiotics, overuse of injections when oral formulations are more appropriate, unreasonable use of expensive drugs, use of brand drug instead of generic drugs, prescription of drugs against clinical guidelines, and self-medication [3, 10–13].

The consequences of irrational prescribing include delays in proper diagnosis and treatment, reduced drug effects, increased side effects, drug resistance, elimination of sources of medicines, increased out-of-pocket payments, dissemination of misconceptions in the common societal culture, reduced patient confidence in the healthcare system, prolonged disease state and even mortality in chronic diseases such as diabetes, hypertension, epilepsy and neurological disorders [3, 4, 8, 14]. Excessive and inappropriate use of antibiotics is a serious threat to the spread of antibiotic resistant bacteria and blood borne infections such as hepatitis and HIV that are transmitted by unsafe injections [4, 8].

Medicines are used less rationally in developing countries than other countries. For example, in Sudan, the use of antibiotics and injections is high but the use of generic drugs is low [9]. In China, 2,500,000 patients per year are strongly influenced by drug side effects. Other problems documented in China, especially in underdeveloped rural areas, include misuse of antibiotics, overuse of injections, inadequate treatment of serious diseases and self-medication [8].

The inappropriate use of drugs due to irrational prescriptions is a common problem in Iran and needs to be controlled [11]. The per capita use of medicines in Iran is high due to drug use culture and the relative cheapness of products. Use is four times the global average [15]. Studies in Iran have shown irrational use of medicines [10, 16–18], but none of them have used the IRDP. This study focused specifically on rural physicians, where access to healthcare is limited and the family physician is the main source of service provision and all prescribed drugs from essential drug list are covered by the insurers. Therefore, they may have a different prescribing pattern than other doctors. A family physician program started in 2005 to promote health and improve the referral system in rural areas and cities with less than 20,000 populations in Iran. Here, 18% of the program’s budget is allocated for the preparation of pharmaceutical items. The family physician framework outlines controlled drug use.

Inappropriate use of drugs is likely to cause harm to both patients and health systems and therefore, policymakers and physicians should understand it and try to reduce it [19]. The key steps for correcting and limiting the irrational use of drugs are to determine the type, amount, and reasons for irrational use [5]. Monitoring the prescribing pattern is the first step. One can then identify appropriate strategies for intervention. Studies of prescribing patterns can provide valuable information about the prescribing behavior of physicians and indicators of irrational drug use. This is useful to managers and policy makers. Therefore, this study explored the prescribing pattern and rational drug use indicators of rural family physicians in Kermanshah province, Iran.

## Methods

This retrospective study was based on performance data from family physicians of Kermanshah province in 2016–2017 (21 Mar 2016–20 Mar 2017). Physicians’ prescriptions data (352,399 prescriptions) were obtained from the information system of the Food and Drug Administration of Kermanshah University of Medical Sciences. Thus, the data had high accuracy.

Kermanshah province has a population of over 1,952,000 people and is located in Western Iran with a rural population of 478,000 [20]. The population lives in 14 counties and has 184 family physicians (102 males and 82 females) in 103 PHC centers. Each PHC center has one or two family physicians that can visit and examine the patients, prescribe medicines and in some cases, refer them to the hospital.

Data analysis was performed using rational use indicators suggested by the WHO [6]: percentage of prescriptions containing antibiotics (optimal: below 30%), percentage of prescriptions containing injection (optimal: below 10%), percentage of drugs prescribed by generic name (optimal: 100%), percentage of drugs prescribed from national essential medicine list (optimal: 100%), and the average number of drugs per prescription (optimal: below 4 items).

Rational drug use was assessed using a measure called Index of Rational Drug Prescribing (IRDP) that has been developed and used in previous studies [3, 4, 8, 9]. Five indicators are used to calculate the IRDP. The optimal level of all five indicators is 1. Values closer to 1 indicate more rational use. Rational use of antibiotics index was calculated by dividing the optimal level (30%) by the percentage of prescriptions containing an antibiotic. The safety injection index was calculated by dividing the optimal level (10%) by the percentage of prescriptions containing an injectable drug. The generic name and essential medicine list indexes were measured via the percentage of drugs prescribed by generic name and the percentage of drugs prescribed from the national essential medicines list. A polypharmacy index was calculated by the percentage of prescriptions with less than four drugs. Finally, the IRDP was calculated by compounding all five prescribing indexes.

According to the Central Bank, the Iranian Rial (IRR) exchange rate to the dollar was (1.00 USD = 32,422 IRR) in 2016–17 [21]. Data analysis was performed using the SPSS.21, and findings were reported as percentages, means, and standard deviations.

## Results

Based on our findings, the average number of drugs per prescription was 3.14 (± 1.2) and the average cost per prescription was 116,740 IRR (USD 3.6). The results of irrational medicine use are shown in Table 1. Fifteen percent of prescriptions had one drug, and around 18.8% had more than four drugs. The maximum of drugs in a prescription was 12. The percentage of prescriptions involving antibiotics and injections was 52.1% and 24.4%, respectively; 95.1% of drugs were generic, and 95.9% were from the essential drugs list. The most prescriptions with antibiotics (60%) and with injection (29.8%) were from Sarpol-e Zahab and Gilan-e Gharb counties, respectively (Table 1).

**Table 1:** Indicators of irrational drug prescribing in rural PHC centers across Kermanshah province

Variables	PCHs (N)	Physicians (N)	average number of drugs per prescription	% prescription with 4 and more drugs	% of prescriptions containing injection	% of prescriptions containing antibiotics	% of drugs prescribed by generic name	% of drugs prescribed from national essential medicine list
Counties								
Eslamabad-e Gharb	9	15	2.8	16.2	22.7	54.8	99.3	98.1
Paveh	10	12	2.3	12.7	29.1	46.0	94.8	95.6
Salas-e Babajani	5	8	1.9	6.5	24.2	51.2	95.8	96.8
Javanrud	4	6	2.7	14.8	27.6	46.8	99.7	96.7
Dalahu	6	11	2.8	17.5	24.2	51.7	94.6	95.6
Ravansar	5	13	2.2	8.5	26.1	47.3	90.1	91.2
Sarpol-e Zahab	9	12	3.9	31.0	25.8	60.6	96.7	97.8
Sonqor	12	15	3.5	24.6	24.9	51.6	99.7	97.5
Sahneh	7	10	3.4	17.0	23.4	38.7	99.7	98.7
Qasr-e Shirin	2	6	2.7	16.2	25.8	47.9	81.2	82.2
Kermanshah	18	47	3.4	19.2	25.1	55.2	94.6	96.7
Kangavar	6	7	3.1	22.4	20.4	54.5	95.1	94.9
Gilan-e Gharb	6	13	3.9	28.3	29.8	52.7	92.4	93.5
Harsin	4	9	4.1	32.2	27.4	55.1	93.8	95.0
Total	103	184	3.14	18.8	24.4	52.1	95.1	95.9

We found that 32.7% of drugs were prescribed in injectable form, which includes piroxicam, dexamethasone, betamethasone and penicillin. Also, 66% of drugs were prescribed in oral form including 31.6% in the form of tablets such as metronidazole, cefixime and adult cold; 26.2% were syrups such as diphenhydramine, and 8.3% were capsules such as amoxicillin and azithromycin.

As shown in Tables 2, 14 counties of Kermanshah province were ranked based on the IRDP. Here, Sahneh at 4.02 had the best performance in terms of rational drug use. Salas-e Babajani and Javanrud are ranked second and third, respectively. The IRDP of Kermanshah’s family physicians was 3.70 out of 5; that was the sum of rational use of antibiotic (0.57), safe injection (0.41), polypharmacy (0.81), generic name (0.95) and essential medicine list (0.96).

**Table 2:** IRDP index in rural family physicians of Kermanshah province

Indexes	Polypharmacy index	Safe injection index	Rational use of antibiotics index	Generic name index	Essential medicine list index	IRDP	Rank
Counties							
Sahneh	0.83	0.43	0.77	1	0.99	4.02	1
Salas-e Babajani	0.94	0.41	0.57	0.96	0.97	3.85	2
Javanrud	0.85	0.36	0.64	1	0.97	3.82	3
Eslamabad-e Gharb	0.84	0.44	0.54	0.99	0.98	3.79	4
Paveh	0.87	0.34	0.65	0.95	0.96	3.77	5
Ravansar	0.92	0.38	0.63	0.90	0.91	3.74	6
Kangavar	0.78	0.49	0.55	0.95	0.95	3.72	7
Dalahu	0.82	0.41	0.57	0.95	0.96	3.71	8
Sonqor	0.75	0.40	0.58	1	0.97	3.70	9
Kermanshah	0.81	0.40	0.54	0.95	0.97	3.67	10
Sarpol-e Zahab	0.69	0.38	0.49	0.97	0.98	3.51	11
Qasr-e Shirin	0.84	0.38	0.63	0.82	0.82	3.49	12
Gilan-e Gharb	0.72	0.33	0.57	0.92	0.93	3.47	13
Harsin	0.68	0.35	0.54	0.94	0.95	3.46	14
Total	0.81	0.41	0.57	0.95	0.96	3.70	–

## Discussion

This study investigated the prescribing patterns of rural family physicians in Kermanshah province. The results showed that the average number of drugs prescribed per prescription was 3.14, which is higher than the recommended optimum level (≤ 3). Studies in Iran have reported this index to be 2.6 to 4.11 [10, 15–17, 22, 23]. In a national study, the average number of drugs per prescription was reported to be 3.07 (for all specialties) and 3.3 for general practitioners. This is close to our findings [11]. This index was also reported in studies conducted in the Eastern Mediterranean Region 2.7 [24], India 2.58 [25], Sudan 2.55 [9], Egypt 2.5 [26], Saudi Arabia 2.4 [3], China 2.36 [8] and in Ethiopia 2.2 [13] that were lower than our study. The average cost per prescription was 116,740 IRR. This was 35,009 in 2010 [23] and 40,903 in 2011 [11] that indicated a three-fold increase in the cost of medicines over the last five years. This could be the result of a leap in inflation between 2011 and 2014.

The polypharmacy index was 0.81, which is relatively good, but polypharmacy can increase drug side effects, patient dissatisfaction, and drug interactions. This results in discontinuation of therapy or a prolonged treatment process [4]. The average number of drugs prescribed per prescription is affected by the burden of diseases, the lack of clinical practice guidelines, the financial incentives for prescribers, the lack of continuous medical education for physicians, physician incompetence, culture, etc. [5]. Thus, different values have been reported in different parts of the world. Unnecessarily prescribed drugs may lead to financial implications for the health system. Rational drug use can prevent wasting medicine and could minimize adverse effects on the patient while reducing costs [3, 26].

Antibiotics were prescribed in 52.1% of prescriptions, which is higher than the optimal level suggested by the WHO (≤ 30%). The results of a national Iranian study showed that 45% of patients received antibiotics [11]. This difference may be due to the regional pattern of diseases. For example, the highest incidence rate of human brucellosis in Iran was related to Kermanshah province [27]. Moreover, the results of a study indicated that the trend of antibiotic consumption in Iran is increasing [28]. The index was 48.9% in Pakistan [4], 48.4% in China [8], 46.8% in Africa [7], 53.6% in the Eastern Mediterranean [24] and 54.7% in Sudan [9], which concur with our study. The rational antibiotic prescription index was also 0.57, which indicates that the degree of rational antibiotic prescription is much lower than the optimal (1.0). Increased antibiotic resistance resulting from overuse is a major worldwide problem [29]. The irrational use of antibiotics can lead to adverse reactions, antibiotic-resistant hospital admissions, and high costs [3, 8, 26]. However, judging the irrationality of antibiotic prescription is difficult because it may be due to differences in local disease patterns.

The percentage of prescriptions containing injections was 24.4%, and the value of safe injection was 0.41. This indicated overuse of injectable drugs. The rate of prescription with injections was 27.1% in the Eastern Mediterranean region [24], 27.1% in Pakistan [4] and 22.9% in China [8] -values that are similar to our findings. Excessive use of injections can increase the risk of blood-related infections such as HIV and hepatitis [8, 26]. Moreover, the costs of injection drugs are always higher than oral medicines, so overuse of injectable drugs when oral forms are more appropriate is irrational drug use [3]. Therefore, steps should be taken to reduce the use of injection, and previous studies have shown that interventions such as training physicians about safe injection can reduce the incidence of injection drug overuse [8].

The use of injections and antibiotics is influenced by several factors including cultural beliefs. A study in Nigeria showed that people believe that injectable drugs are more effective than oral drugs. The needle and syringe is a symbol of Western medicine in the developing world [30]. This belief is also found in Iranian society and can explain the motivation for injectable drugs. The people—especially in rural areas—believe that injections provide quicker and more complete pain relief than oral forms [4].The patient’s demand and request for faster treatment is the most important motive for the doctors to prescribe an injectable drug. Insufficient training and financial incentives for physicians are other factors [31].

The value of the essential medicine list index was 0.96. In other words, 96% of the drugs prescribed were from the list, which is quite close to the ideal value (100%). Other values include 67.7% in China [8], 81.2% in Sudan [9], 88% in the Africa [7], 90.6% in Tanzania [32], 91% in the Eastern Mediterranean region [24], 93% in Pakistan [4] and 92% Ethiopia [13]. This indicates that family physicians in Kermanshah performed better in this indicator. This might be because of drug legislation. The Iran Medicine List (IML) is the main base for pharmaceutical activities and prescriptions outside of the IML are banned by national law [33]. Also, in the Iran family physician program, physicians are not allowed to prescribe outside of the list, otherwise their performance bonus will be reduced. The WHO strongly recommends that prescriptions from the essential medicine list. Drugs on the list are older, practically tested, with established clinical use, and at lower cost than newer drugs [3, 26].

We found that 95% of drugs were generics. In other regions these values are: Sudan 46.3% [9], Eastern Mediterranean 57.1% [24], Saudi Arabia 61.2% [3], China 64.1% [8], Africa 68% [7], Pakistan 71.6% [4] and Tanzania 85.6% (27). These all indicate that family physicians in Kermanshah was superior in using generics. The Iranian drug selection committee provides a list of generic name drugs for the IML and therefore prescription is based on generic names [33]. Medical decisions are based physicians’ personal beliefs, extensive promotional activities by pharmaceutical companies, and a lack of adherence to regulations for prescribing generic drugs, all can affect this indicator [4]. The WHO highly recommends prescribing generic drugs for safety because it enables easy exchange of information and better communication between health care providers [3, 26].

The IRDP was 3.70 compared to an optimal level of 5. This shows that 74% of the rational drug-prescribing standards were met. This was 3.32 in China [8], 3.39 in Sudan [9] and 3.42 in India [34]. These values were lower than in our study. Some of the factors affecting the irrational prescribing include a culture of drug use, work environments, medicine supply, laws and regulations, lack of information or misinformation about drugs, prescribing habits of physicians, improper monitoring by regulatory entities, insufficient continuing education, demands of patients, and financial benefits from selling drugs [1, 4, 35].

### Limitations

This study was conducted in only one province. However, the family physician model is used in all rural areas of Iran, and it can be assumed that the pattern of drug prescription is similar throughout Iran. This study did not seek to understand the reasons for irrational use, but this can be explored in future studies.

## Conclusion

Analysis of drug prescription patterns can provide valuable information about potential problem areas in drug use to managers and policymakers. They can use the results to develop appropriate interventions to improve prescribing. The findings showed that some degrees of irrational drug use exists among family physicians—especially in terms of prescribing injections and antibiotics and polypharmacy. The extent to which drug consuming is irrational not easily judged. It should be judged by considering the socio-economic characteristics of a region, as well as the diseases pattern.

Based on these findings, we recommend retraining and continuing education for physicians, residents and pharmacists regarding rational use of antibiotics and injections as well as drugs for medical indications. The use of clinical practice guidelines will help increase the rational use of medicines. In addition, we also suggest efficient and continuous monitoring of drug use.

## Abbreviations

IML: Iran Medicine List
INRUD: International Network for Rational Use of Drugs
IRDP: Index of Rational Drug Prescribing
IRR: Iranian Rial
PHC: Primary Health Care
WHO: World Health Organization

## References

[1] Ofori-Asenso R, Agyeman AA (2016). Irrational use of medicines—a summary of key concepts. Pharmacy.

[2] Wirtz VJ, Hogerzeil HV, Gray AL, Bigdeli M, de Joncheere CP, Ewen MA, Gyansa-Lutterodt M, Jing S, Luiza VL, Mbindyo RM (2017). Essential medicines for universal health coverage. Lancet.

[3] El Mahalli A (2012). WHO/INRUD drug prescribing indicators at primary health care centres in eastern province. Saudi Arabia East Mediterr Health J.

[4] Atif M, Sarwar MR, Azeem M, Naz M, Amir S, Nazir K (2016). Assessment of core drug use indicators using WHO/INRUD methodology at primary healthcare centers in Bahawalpur, Pakistan. BMC Health Serv Res.

[5] Atif M, Sarwar MR, Azeem M, Umer D, Rauf A, Rasool A, Ahsan M, Scahill S (2016). Assessment of WHO/INRUD core drug use indicators in two tertiary care hospitals of Bahawalpur, Punjab, Pakistan. J Pharm Policy Pract.

[6] Ofori-Asenso RA (2016). Closer look at the World Health Organization's prescribing indicators. J Pharmacol Pharmacother.

[7] Ofori-Asenso R, Brhlikova P, Pollock AM (2016). Prescribing indicators at primary health care centers within the WHO African region: a systematic analysis (1995–2015). BMC Public Health.

[8] Dong L, Yan H, Wang D (2011). Drug prescribing indicators in village health clinics across 10 provinces of western China. Fam Pract.

[9] Yousif BME, Supakankunti S (2016). General practitioners’ prescribing patterns at primary healthcare centers in National Health Insurance, Gezira, Sudan. Drugs Real World Outcomes.

[10] Ahmadi B, Arab M, Narimisa P, Janani L, Najafpour J (2013). Surveying prescription pattern medication family physician and capitation drug in Ahwaz. J Healthc Manag.

[11] Karimi A, Haerizadeh M, Soleymani F, Haerizadeh M, Taheri F (2014). Evaluation of medicine prescription pattern using World Health Organization prescribing indicators in Iran: a cross-sectional study. J Res Pharm Pract.

[12] Summoro TS, Gidebo KD, Kanche ZZ, Woticha EW (2015). Evaluation of trends of drug-prescribing patterns based on WHO prescribing indicators at outpatient departments of four hospitals in southern Ethiopia. Drug Des Devel Ther.

[13] Bilal AI, Osman ED, Mulugeta A (2016). Assessment of medicines use pattern using World Health Organization’s prescribing, patient care and health facility indicators in selected health facilities in eastern Ethiopia. BMC Health Serv Res.

[14] Yousefi N, Majdzadeh R, Valadkhani M, Nedjat S, Mohammadi H (2012). Reasons for physicians’ tendency to irrational prescription of corticosteroids. Iran Red Crescent Med J.

[15] Hosseinzadeh F, Sadeghieh Ahari S, Mohammadian-erdi A (2016). Survey the antibiotics prescription by general practitioners for outpatients in Ardabil City in 2013. Journal of Ardabil University of Medical. Sciences..

[16] Khadivi R, Yarahmadi A (2014). The drug prescription patterns and utilization after family physician program implementation in rural health centers of Isfahan District. Iran Journal of Isfahan Medical School.

[17] Zareshahi R, Haghdoost A, Asadipour A, Sadeghirad B (2012). Rational usage of drug indices in the prescriptions of kerman medical practitioners in 2008. Journal of Rafsanjan University of Medical Sciences.

[18] Vali L, Pourreza A, Rahimi FA, A Sari A, H Honarmand P (2012). An InvestigationOn inappropriate medication applied among elderly patients. World Applied Sciences Journal.

[19] Brownlee S, Chalkidou K, Doust J, Elshaug AG, Glasziou P, Heath I, Nagpal S, Saini V, Srivastava D, Chalmers K. Evidence for overuse of medical services around the world. Lancet 2017, 390(July):156–168. 10.1016/S0140-6736(16)32585-5 PMC5708862 28077234

[20] Statistical Centre of Iran. In: General Census of Population and Housing 2016. https://www.amar.org.ir.

[21] Centeral Bank of Iran. In: Exchange rates. http://www.cbi.ir/exrates/rates_fa.aspx.

[22] Arab M, Torabipour A, Rahimifrooshani A, Rashidian A, Fadai N, Askari R (2014). Factors affecting family physicians’ drug prescribing: a cross-sectional study in Khuzestan, Iran. Int J Health Policy Manag.

[23] Safaeian L, Mahdanian A-R, Hashemi-Fesharaki M, Salami S, Kebriaee-Zadeh J, Sadeghian G-H (2011). General physicians and prescribing pattern in Isfahan. Iran Oman Med J.

[24] Holloway K, Ivanovska V, Wagner A, Vialle-Valentin C, Ross-Degnan D (2013). Have we improved use of medicines in developing and transitional countries and do we know how to? Two decades of evidence. Tropical Med Int Health.

[25] Banerjee I, Bhadury T (2014). Prescribing pattern of interns in a primary health center in India. J Basic Clin Pharm.

[26] Akl OA, El Mahalli AA, Elkahky AA, Salem AM (2014). WHO/INRUD drug use indicators at primary healthcare centers in Alexandria, Egypt. Journal of Taibah University Medical Sciences.

[27] Mirnejad R, Jazi FM, Mostafaei S, Sedighi M: Epidemiology of brucellosis in Iran. A comprehensive systematic review and meta-analysis study. Microb Pathog2017, 109(Aug):239–247. 10.1016/j.micpath.2017.06.005 28602839

[28] Abdollahiasl A, Kebriaeezadeh A, Nikfar S, Farshchi A, Ghiasi G, Abdollahi M (2011). Patterns of antibiotic consumption in Iran during 2000–2009. Int J Antimicrob Agents.

[29] Yin X, Song F, Gong Y, Tu X, Wang Y, Cao S, Liu J, Lu ZA (2013). Systematic review of antibiotic utilization in China. J Antimicrob Chemother.

[30] Adebayo E, Hussain N (2010). Pattern of prescription drug use in Nigerian army hospitals. Ann Afr Med.

[31] Cheraghali AM, Solemani F, Behmanesh Y, Habibipour F, Ismaeilzadeh A, Nikfar S, Rahimi W (2006). Physicians' attitude toward injectable medicines. J Pharmacol Toxicol.

[32] Mambile G, Konje E, Kidenya BR, Katabalo D, Marwa K (2016). Quality of drug prescription in primary health care facilities in Mwanza, north-western Tanzania. Tanzania Journal of Health Research.

[33] Zargaran M, Nikfar S, Cheraghali AM (2016). Evaluation of prescriptions of medicines not included in Iran medicine list: a cross-sectional study. J Res Pharm Pract.

[34] Kasabi GS, Subramanian T, Allam RR, Grace CA, Reddy S, Murhekar MV (2015). Prescription practices & use of essential medicines in the primary health care system, Shimoga district, Karnataka. India Indian J Med Res.

[35] Mousavi S, Zargarzadeh A (2015). Rational drug use in Iran: a call for action. Journal of Pharmaceutical Care.

